# Targeting HER-3 to elicit antitumor helper T cells against head and neck squamous cell carcinoma

**DOI:** 10.1038/srep16280

**Published:** 2015-11-05

**Authors:** Takumi Kumai, Takayuki Ohkuri, Toshihiro Nagato, Yoshinari Matsuda, Kensuke Oikawa, Naoko Aoki, Shoji Kimura, Esteban Celis, Yasuaki Harabuchi, Hiroya Kobayashi

**Affiliations:** 1 Department of Pathology, Asahikawa Medical University, Asahikawa, Japan; 2 Department of Otolaryngology, Head and Neck Surgery, Asahikawa Medical University, Asahikawa, Japan; 3 Cancer Immunology, Inflammation and Tolerance Program, Georgia Regents University Cancer Center, Augusta, GA.

## Abstract

HER-3 expression has been reported to act as an important oncoprotein in head and neck squamous cell carcinoma. This protein is known to control tumor proliferation and acquisition of resistance by tumor cells towards EGFR inhibitors, therefore, development of a HER-3-targeted therapy is desirable. In this study, we found that HER-3 expression on tumor cells was increased after EGFR inhibition. To establish a novel therapeutic approach for HER-3-positive head and neck carcinoma, we identified a HER-3 helper epitope that could elicit effective helper T cell responses to the naturally processed HER-3-derived epitope presented in a HER-3 expressing tumors. This epitope induced potent cytolytic activity of CD4 T cells against such tumor cells. Moreover, pan HER-family tyrosine kinase inhibitor augmented the responses of HER-3-reactive CD4 T cells via upregulation of HLA-DR protein on the surface of tumor cells. Our results supports the validity of CD4 T cell-dependent HER-3-targeted therapy combined with a broad inhibitor of HER-family.

Targeting therapy to the epidermal growth factor receptor (EGFR) is a new and effective treatment for head and neck squamous cell carcinoma (HNSCC). Several EGFR tyrosine kinase inhibitors and anti-EGFR therapeutic antibodies have been used in clinical studies and have shown therapeutic effects against locally advanced, recurrent, or metastatic HNSCC^1^,^2^. Nevertheless, both acquired and innate resistance reduces the efficacy of these therapeutic agents^3^,^4^. Feedback activation of the alternative pathway by which a tumor can proliferate under EGFR inhibition is one of the mechanisms of acquired drug resistance of HNSCC to EGFR inhibitors^5^. Among these alternative pathways, HER-3 signaling is believed to play a considerable role in the proliferation of tumors that are treated with EGFR inhibitors^6^,^7^. HER-3 is a member of the ErbB receptor tyrosine kinase family can function as an oncoprotein in solid tumors, binding ligands such as neuregulins, inducing EGFR/HER-3 and HER-2/HER-3 heterodimers that activate downstream signaling pathways^8^. Because HER-3 overexpression is associated with high mortality in HNSCC, targeting HER-3 would be expected to provide a therapeutic benefit^9^,^10^. However, conventional inhibitors of ErbB family tyrosine kinases cannot inhibit HER-3 activity because HER-3 by itself does not have a tyrosine kinase activity. Hence, novel alternative therapeutic approaches such as T cell based immunotherapy could be used to target HER-3.

Previously, we reported that the T helper cell epitope EGFR_875−889_ bears high amino acid sequence homology with an analogous portion of the HER-3 protein^11^. Furthermore, EGFR_875−889_-reactive helper T cells cross-reacted with the HER-3 peptide analog. However, it remained unclear whether the HER-3 analog peptide was able to induce T-cell responses capable of recognizing HER-3-expressing tumors. In the present study, we demonstrate that this HER-3 peptide analog was effective in inducing HER-3-reactive CD4 T cells that directly recognize and kill HNSCC cells. In addition, we found that a broad inhibitor of the HER family augmented helper T-cell responses against the tumor cells via HLA-DR upregulation. These results indicate that targeting HER-3 as a tumor associated antigen (TAA) together with HER-targeted inhibitors could be an effective approach to treat HNSCC.

## Results

### Cell surface expression of HER-3 is upregulated by a broad HER family inhibitor

**Figure 1:**
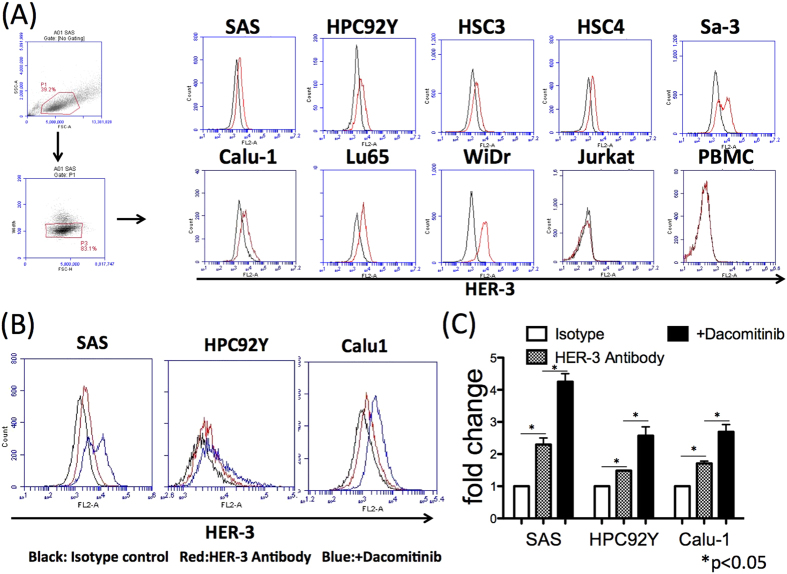
HER-3 upregulation on the surface of tumor cells by a pan HER family inhibitor. (**A**) HER-3 expression on tumor cells was assessed using flowcytometry. Head and neck squamous cell carcinoma (HNSCC) cell lines (SAS, HPC92Y, HSC3, HSC4, and Sa-3), lung cancer cell lines (Calu-1 and Lu65), and a colon cancer cell line (WiDr) cell lines expressed HER-3. Jurkat cells and peripheral blood mononuclear cells (PBMCs) from healthy donor were used as negative controls. The dead cells and the doublets were eliminated by FSC/SSC gating before the analysis. Black lines: Isotype control; Red lines: anti-HER-3 antibody. (**B**,**C**) HER-3 expression on SAS, HPC92Y and Calu-1 cells was evaluated after 48-h pre-incubation with dacomitinib (1 μM). Black: isotype control, red: an anti-HER-3 antibody, blue: addition of dacomitinib. Mean fluorescence intensity (MFI) fold changes in the isotype antibody group are shown in the graph: *p < 0.05. Columns: means of triplicate measurements, error bars: SEM. The dead cells and the doublets were eliminated by FSC/SSC gating before the analysis. The results shown are representative of 3 separate experiments.

It has been reported that HER-3 plays a significant role in the development of EGFR inhibitor resistance in tumors^7^. Because the efficacy of EGFR inhibitors in the treatment of HNSCC is partly reduced by acquired resistance, targeting HER-3 could be a promising strategy for patients who become refractory to EGFR inhibitors. Thus, we first measured the surface expression of HER-3 by HNSCC, lung cancer, and colon cancer cell lines. All the solid tumor cell lines expressed HER-3 on the cell surface, while Jurkat T cell lymphoma and PBMCs did not (Fig. 1A). When 3 of these tumor cell lines (SAS, HPC9Y and Calu-1) that expressed low levels of HER-3 were treated with an irreversible HER family broad inhibitor (dacomitinib, which inhibits EGFR, HER-2 and HER-4 but not HER-3), the expression of HER-3 was substantially increased (Fig. 1B,C). HER-3 expression on the other cell lines that strongly expressed high level of HER-3 and on negative-control cells (Jurkat and PBMCs) was not affected by dacomitinib (data not shown). These results support the possibility of targeting HER-3 as an antigen for cancer immunotherapy, especially after or during therapy with HER family broad inhibitors.

### Induction of HER-3-reactive CD4 T cells.

**Figure 2:**
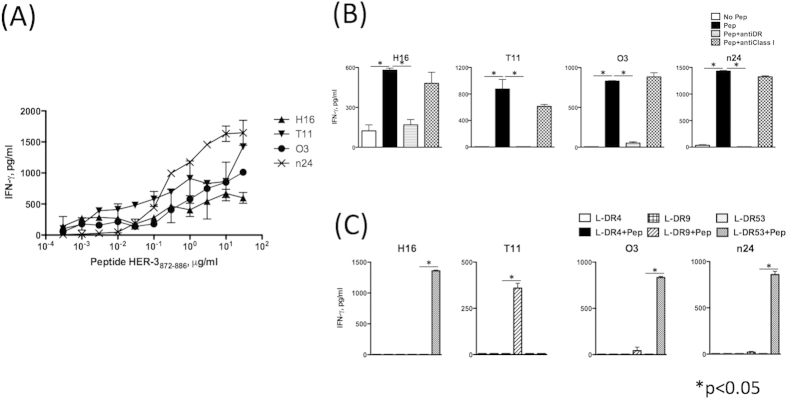
Induction of HER-3-reactive CD4 T cells. (**A**) HER-3-reactive CD4 T cells were elicited in 4 healthy individuals (H16 is from donor1: DR4/9, DR53; T11 is from donor2: DR9/12, DR53; O3 is from donor3: DR9/13, DR53; n24 is from donor4: DR9/13, DR53) and then tested for their ability to produce IFN-γ in response to HER-3 peptides. Peptides (0–30 μg/ml) were loaded on irradiated autologous PBMCs. Points: means of triplicate measurements, bars: SEM. The results shown are representative of 3 experiments that were performed on the same samples. (**B**) We used an IFN-γ assay to assess the responses of HER-3-reactive CD4 T cells to HER-3 peptide (3 μg/ml)-loaded irradiated autologous PBMCs. L243 (10 μg/ml, an anti-HLA-DR antibody) or W6/32 (10 μg/ml, an anti-HLA Class I antibody) was added to confirm the HLA-DR restriction of the T-cell responses. (**C**) L-DR4, L-DR9 or L-DR53 cells with or without HER-3 peptide (3 μg/ml) were used as antigen-presenting cells (APCs) to evaluate the specific HLA-DR restriction of each HER-3-reactive CD4 lines by means of an IFN-γ assay. Columns: means of triplicate measurements, bars: SEM. The results shown are representative of 3 separate experiments.

We previously identified a CD4 T cell peptide epitope in the EGFR protein that was effective in inducing antitumor responses^11^. Interestingly, CD4 T cells induced with this EGFR peptide epitope, (EGFR_875−889_, KVPIKWMALESILHR) were capable of recognizing the corresponding HER-3_872−886_ peptide (KTPIKWMALESIHFG), which bears 73% amino acid homology. The CD4 T cells also recognized EGFR-negative tumors that expressed HER-3. In view of these findings, we proceeded to test whether the HER-3_872−886_ peptide itself could induce antigen-specific, tumor-reactive CD4 T cells. Stimulation of CD4 T cells, purified from PBMCs of 4 healthy donors with HER-3_872−886_-pulsed autologous DCs resulted in the induction of peptide-reactive CD4 T cell lines that were subsequently cloned. As shown in Fig. 2A, four T cell lines secreted IFN-γ when stimulated with the peptide antigen in a dose dependent manner. The analysis of T cell receptor Vβ usage showed that these cell lines were heterogenous populations but the majority of each cell line was consisted of single Vβ positive cells (Supplementary Fig. 1). Production of IL-4, IL-5, IL-10 and IL-17A was not detected in the peptide-stimulated T cell lines (not shown). The addition of an anti-HLA-DR antibody to the peptide stimulation assays resulted in the inhibition of IFN-γ production by HER-3 reactive CD4 T cell lines indicating that peptide HER-3_872−886_ was recognized in the context of MHC class II molecules and specifically by HLA-DR since this antibody (L243) does not react with the HLA-DQ and -DP proteins (Fig. 2B). As expected, peptide recognition by the CD4 T cell lines was not affected by the addition of anti-HLA class I antibody (W6/32). To determine the HLA-DR alleles restricting the responses in these T cell lines, we used a panel of mouse fibroblasts expressing single HLA-DR molecules (L-DR4, L-DR9, L-DR53) as APCs. The results shown in Fig. 2C indicate that CD4 T cell lines H16, O3, and n24 recognized the HER-3_872−886_ in the context of HLA-DR53, whereas T11 was restricted by HLA-DR9. Because the frequency of HLA-DR53-linked HLA-DR alleles has been reported to be 39% in the HNSCC patients^12^, these results indicate that peptide HER-3_872−886_ is able to elicit CD4 responses in broad population of HNSCC patients.

### Antitumor responses mediated by HER-3-reactive CD4 T cells

**Figure 3:**
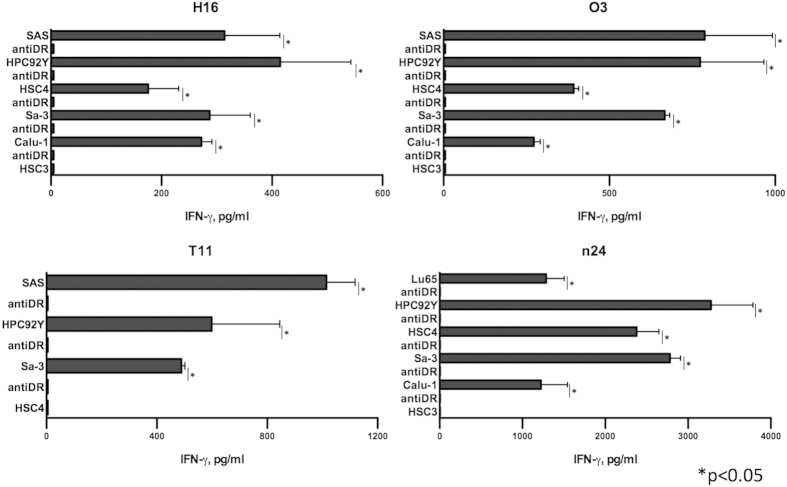
Direct recognition of tumor cells by HER-3-reactive CD4 T cells. The HLA-DR-53-restricted (H16, O3, and n24) and DR-9-restricted (T11) HER-3-reactive CD4 T cells were tested for their ability to directly recognize HLA-DR-matched tumor cells. HSC3 cells (DR15/15) or HSC4 cells (DR1/4, DR53) were used as an HLA-DR-unmatched negative control for DR9- or DR53-restricted HER-3-reactive CD4 T cells, respectively. L243 cells were used to verify the HLA-DR restriction of these responses. Columns: means of triplicate measurements, bars: SEM. The results shown are representative of 3 separate experiments.

**Figure 4:**
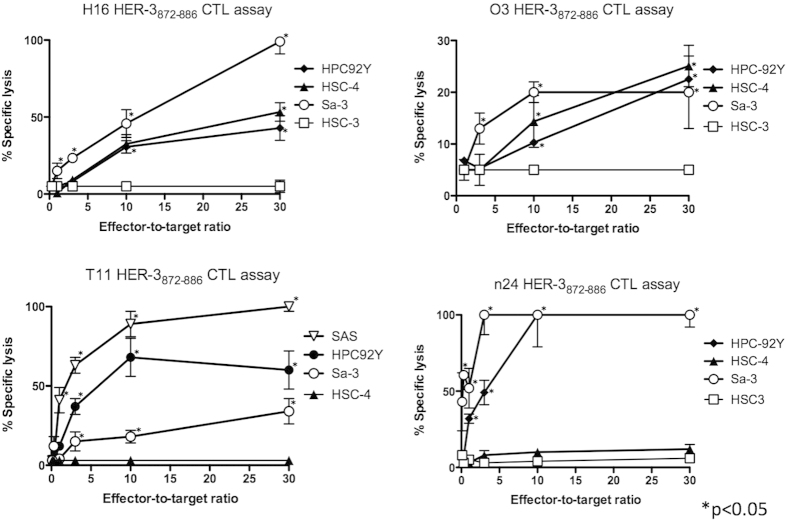
Cytotoxicity of HER-3-reactive CD4 T cells toward tumor cells. HER-3-reactive CD4 T cells were tested for their ability to lyse tumor cells at various effector-to-target ratios. HLA-DR-unmatched tumor cell lines (HSC3 or HSC4 cells) served as a negative control. The results shown are representative of 3 experiments that were performed on the same samples. The p-value of each plot was calculated by comparing with the specific tumor lysis in the absent of T cells (Effector-to-target ratio = 0).

Although the HER-3 protein is expressed on tumor cells, presentation of the HER-3_872−886_ peptide on tumor cells through the endogenous antigen-processing machinery is required for CD4 T cell recognition. To find out whether HER-3_872−886_ peptide is presented on MHC class II molecules on the surface of tumor cells, we cocultured HER-3-reactive CD4 T cells and HLA-DR-matched HER-3-positive tumor cells, and measured IFN-γ production in the culture supernatants. Because IFN-γ is required to induce MHC class II expression on tumor cells^13^ and HER-3-reactive CD4 T cells did not react with IFN-γ untreated tumor (data not shown), tumor cells were pretreated with IFN-γ. The results showed that HER-3-reactive CD4 T cells directly reacted with tumor cells in an HLA-DR-restricted manner (Fig. 3). Because cytotoxicity of CD4 T cells is involved in antiviral and antitumor immunity^14^,^15^, we also evaluated the cytotoxic activity of the HER-3-reactive CD4 T cells against the tumor cells. As shown in Fig. 4, some of these T cell lines were effective in killing HLA-DR-matched HER-3-positive tumor cells. Interestingly, although n24 reacted with the HSC4 tumor cells producing IFN-γ (Fig. 3), these CD4 T cells did not lyse the HSC4 cells. These results indicate that tumor recognition (cytokine production) does not necessarily correlate with lytic function. Because it was possible that readout of this cytotoxicity assay, lactate dehydrogenase (LDH) in the supernatant might be also derived from CD4 T cells due to the coculture with tumor^16^, HER-3-reactive CD4 T cells were stained with Annexin-V and 7-AAD to clarify the source of LDH in the coculture system. As a result, coculture with tumor had no effect on the viability of the HER-3-reactive CD4 T cells suggesting that LDH was only derived from tumor cells in the cytotoxicity assay (Supplementary Fig. 2). By immunohistochemical staining in human tissues, the level of HER-3 expression was significantly higher in the cancer specimens than in normal mucosa membrane (Supplementary Fig. 3). Because a relatively low concentration of antigen has been reported to induce T cell ignorance^17^, it is speculated that HER-3 reactive T-cells cannot trigger detrimental autoimmunity. Taken together, these results suggest that the HER-3_872−886_ epitope could be used to develop antigen-specific CD4 T cell based therapies such as a peptide vaccine for HER-3-positive tumors.

### Recognition of a naturally processed exogenous antigen by HER-3-reactive CD4 T cells

**Figure 5:**
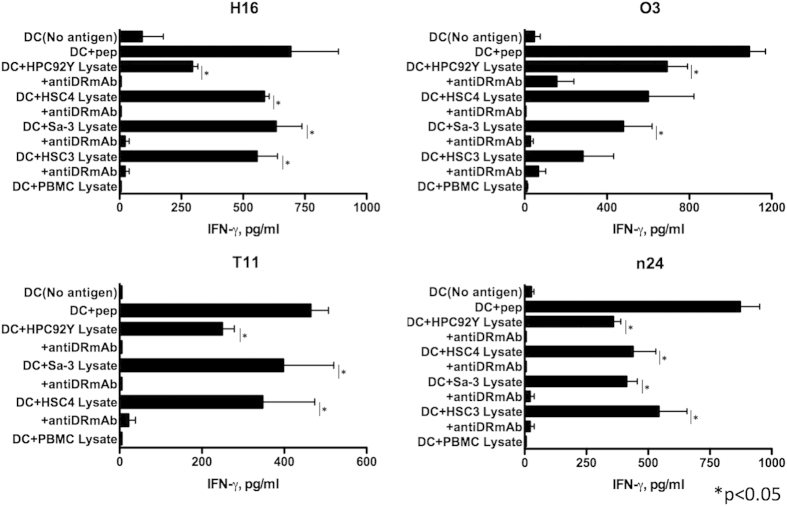
Recognition of a naturally processed exogenous antigen by HER-3-reactive CD4 T cells. The ability of HER-3-reactive CD4 T cells to recognize naturally processed exogenous HER-3 was assessed judging by IFN-γ production. Dendritic cells (DCs) were used as antigen-presenting cells (APCs), and HLA-DR-matched or -unmatched tumor cell lysates served as sources of HER-3 protein. L243 cells were used to verify the HLA-DR restriction of these responses. DC without antigen or pulsed with PBMC lysate was used as a negative control and DC pulsed with HER-3_872−886_ was used as a positive control. Columns: means of triplicate measurements, bars: SEM. The results shown are representative of 3 separate experiments.

We next determined whether the HER-3_872−886_ epitope can be processed from tumor lysates and efficiently presented by MHC class II molecules on DCs. HER-3 reactive CD4 T cells recognized not only HLA-DR-matched tumor cell lysates but also DCs pulsed with HER-3-positive HLA-DR-unmatched tumor cell lysates (Fig. 5). These findings indicate that the exogenous antigen-processing machinery produces the HER-3_872−886_ epitope that is capable of stimulating the CD4 T cells.

### Pan HER-family inhibitor augments tumor recognition by HER-3-reactive CD4 T cells

**Figure 6:**
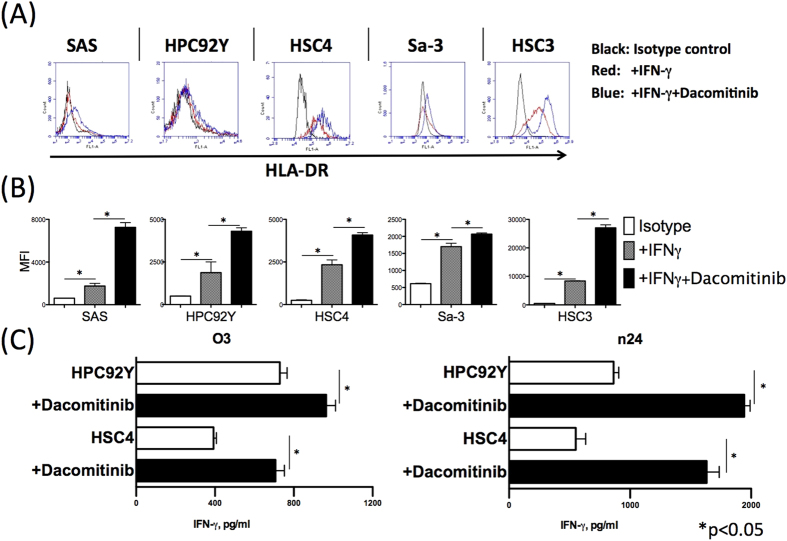
pan HER-family inhibitor augments antitumor responses of HER-3-reactive CD4 T cells. (**A**,**B**) HLA-DR expression on the surface of SAS, HPC92Y, HSC4, Sa-3, and HSC3 cells was evaluated using flow cytometry after 48-h preincubation with IFN-γ (100 IU/ml) alone or with dacomitinib (1 μM) by flowcytometry. Columns: means of triplicate measurements, bars: SEM. The results shown are representative of 3 separate experiments. (**C**) We used an IFN-γ assay to evaluate the responses of HER-3-reactive T cells (O3 and n24) to tumor cells pretreated for 48 h with IFN-γ (100 IU/ml) alone or with dacomitinib (1 μM): n.s.: not significant; *p < 0.05. Columns: means of triplicate measurements, bars: SEM. The dead cells and the doublets were eliminated by FSC/SSC gating before the analysis. The results shown are representative of 3 separate experiments.

We and others reported that HLA-DR expression on tumor cells is augmented by EGFR inhibition^11^,^18^. Thus, we next tested whether dacomitinib (pan HER family inhibitor) can affect HLA-DR expression on tumor cells. After incubation with dacomitinib and IFN-γ for 48 h, tumor cells showed increased surface expression of HLA-DR molecules compared to the group with IFN-γ treatment alone (Fig. 6A,B). We next determined the effects of dacomitinib pretreatment on tumor cell recognition by HER-3-reactive CD4 T cells. As shown in Fig. 6C, pretreatment of the tumor cells with dacomitinib augmented the reactivity of HER-3-reactive CD4 T cells. This result suggests that a pan HER-family inhibitor could be used as an adjuvant for antitumor immunotherapy.

### HER-3 peptide recognition by PBMCs from HNSCC patients

**Figure 7:**
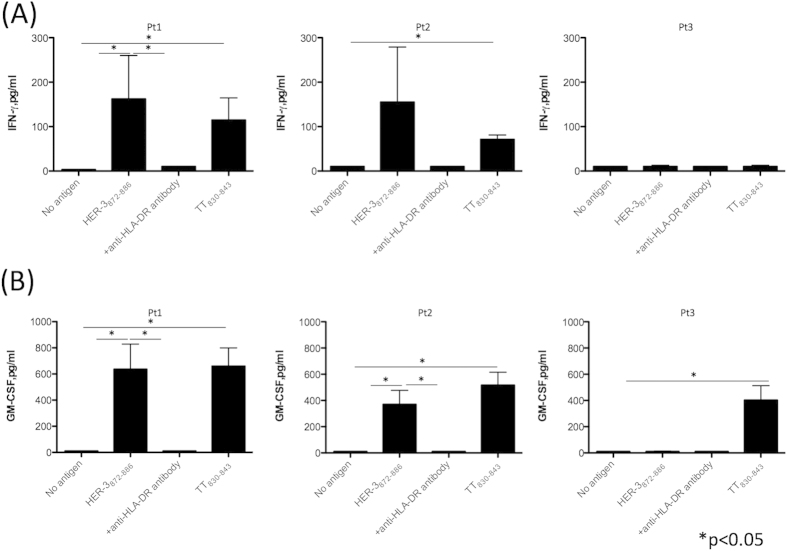
T cell responses to HER-3 peptide in PBMCs from HNSCC patients. PBMCs from three HNSCC patients (Pt1: hypopharyngeal squamous cell carcinoma, Stage IV; Pt2: tongue squamous cell carcinoma, Stage IV, and Pt3: oropharyngeal squamous cell carcinoma, Stage IV) were stimulated with HER-3_872−886_ for seven days. Tetanus toxin_830−843_ was used as a positive control. After culture, PBMCs were re-stimulated with peptide-pulsed irradiated auto PBMCs for another seven days. Culture supernatant was collected to measure the production of IFN-γ (**A**) and GM-CSF (**B**). Columns: means of triplicate measurements, bars: SEM. The results shown are representative of 3 experiments that were performed on the same samples.

It is valuable to determine the presence of precursor T cells specific for HER-3 peptides in the HNSCC patients for confirming the immunogenicity of HER-3 and predicting the potential use of this peptide as a cancer vaccine. To evaluate the CD4 T cell responses to HER-3_872−886_ in HNSCC patients, we carried out a short-term culture using peptide-stimulated PBMCs from three HNSCC patients. These HNSCC patients did not show any clinical signs of autoimmune diseases. Because tetanus toxin peptide has an ability to elicit robust CD4 T cell responses in the great portion of people regardless of the HLA-DR alleles^19^, this peptide was used as a positive control. As shown in Fig. 7, substantial T cell responses to HER-3_872−886_ were observed in two HNSCC patients and these responses were suppressed by anti-HLA-DR mAb suggesting that the responses were mediated through the MHC-II-peptide-T-cell receptor complexes. Taken together, HER-3_872−886_ has the potential to induce T cell responses not only in healthy donors but also in HNSCC patients.

## Discussion

In this study, we have identified a novel HER-3-derived helper CD4 T cell epitope that can induce an effective antitumor responses; we also demonstrated the utility of a pan-HER family inhibitor as an immunotherapy adjuvant. As HER-3 is expressed by all HNSCC cell lines used in this study and the HER-3 expression is known to be linked to HNSCC mortality, this protein seems to be a suitable tumor antigen for developing cancer immunotherapy^9^,^10^. As reported previously, we also detected HER-3 expression in lung and colon cancer cell lines, implicating that HER-3-targeted immunotherapy could be used for lung and colon cancer treatment^20^,^21^. Although it is difficult to simply consider lymphocyte infiltration as a surrogate marker for the immunogenicity of HER-3 because tumor is known to inhibit lymphocyte infiltration by soluble factors (e.g. semaphorin 4D) in most likely TCR-independent manner^22^, we found in the TCGA database that 66% of the HNSCC samples, which have an amplified expression of HER-3 gene, have lymphocyte infiltration^23^. Since the information about the actual expression level of HER-3 protein instead of gene expression is not available in the database, further evaluation is required to confirm the immunogenicity of HER-3 in the tumor microenvironment. Because the frequency of CD8 T cells specific for tumor antigen is higher in HNSCC patients (0.02–0.04%) than in healthy controls^16^, it is probable that HER-3-specific CD4 T cells are also increased in HNSCC patients. While it is difficult to determine the frequency of antigen-specific CD4 T cells because of the lack of reliable method such as tetramer staining to detect these few CD4 T cells, we described that HER-3 reactive CD4 T cells existed in HNSCC patients indicating the immunogenicity of HER-3 in HNSCC patients (Fig. 7).

The concern about targeting tumor-associated antigen is that most of this type of antigens is commonly expressed in the healthy tissues in which autoimmune diseases are developed. Indeed, HER-3 was slightly expressed in normal colon mucosa (Supplementary Fig. 3). However, there are several reasons why targeting HER-3 is still promising and would not cause autoimmune diseases. Firstly, the level of HER-3 expression on normal tissues is significantly lower than cancer. Because T cell has been reported to ignore a relatively low concentration of antigen^17^, HER-3 reactive T-cells would not react with normal tissues. Moreover, HLA-DR expression was not detected in normal mucosa membrane suggesting that normal mucosa is not a suitable target of CD4 T cells (data not shown). Secondly, it may be possible that only low affinity self antigen-reactive T cells that require a high amount of antigen can survive through selection in thymus. Indeed, we showed that HER-3 reactive CD4 T cells existed in HNSCC patients without any clinical signs of autoimmunity (Fig. 7). Similar to HER-3, EGFR is expressed in cancer but also in normal epithelial cells. In HNSCC patients, EGFR-reactive T cell frequencies correlate with EGFR expression in cancer without any clinical features of autoimmunity suggesting that these T cells can recognize tumor as an antigen but not normal tissue in which the amount of EGFR epitope might not be enough to exceed the threshold of TCR signaling^24^. Nevertheless, further studies are required to ascertain the safety of targeting HER-3 (mouse homologue: ErbB3) as an immunogen by using *in vivo* model. The problematic part of the *in vivo* model in immunotherapy is that the epitope that can stimulate T cells is different between each species. Although HLA transgenic mice has a potential to induce mouse T cell responses by human epitopes, most of the HLA transduced in mice are HLA class I. Adoptive cell transfer of HER-3 reactive CD4 T cells into immunocompromised mice might be a option to see the antitumor activity of these cells against human tumor. However, the antitumor effect of CD4 T cells is not only based on the direct cytotoxicity but also its interaction with other immune cells. For example, IFN-γ production from CD4 T cells augments MHC class I expression on tumor followed by CD8 T cells-mediated tumor killing^25^. In addition, immunocompromised model cannot be utilized for the sake of safety because these mice express mouse MHC instead of HLA. Thus, it would be valuable to identify the mouse ErbB-3 (mouse homologue of human HER-3) CD4 epitope to clarify the safety and the efficacy of targeting HER-3 as an antigen for peptide vaccine.

We have shown that the IFN-γ production by CD4 T cells against tumor was not a perfect marker of direct tumoricidal activity of these cells (Figs 3 and 4). Several mechanisms have been proposed to describe the direct antitumor activity of CD4 T cells. Tumor Necrosis Factor–related Apoptosis-inducing Ligand (TRAIL) is upregulated on CD4 T cells and has a cytotoxic activity against renal cell carcinoma^26^. We have previously shown that STEAP-reactive CD4 T cells expressed granzyme B, which is a key element of cellular cytotoxicity by the granule exocytosis pathway^14^. Although IFN-γ production from CD4 T cell may not show a direct antitumor effect, it is still possible that this cytokine can be a prognostic marker *in vivo* because IFN-γ production in tumor microenvironment could upregulate antitumor immunity in an indirect manner by augmenting CD8 T cell reactivity to tumor cells subsequent to MHC class I expression. Thus, further evaluation is required to identify whether IFN-γ production by CD4 T cells can be a reliable marker of antitumor effect in the peptide vaccine therapy.

Cell signaling is a dynamic mechanism utilized by cancer cells to survive under multiple stressors such as chemo-radiotherapy and immune surveillance. After identification of signaling pathways specific to each cancer cell type, signaling molecule-targeted therapy has become one of the pivotal tools for the treatment of cancer^27^. In the case of HNSCC, EGFR is regarded as a promising therapeutic target because of its strong expression; therefore, EGFR-targeting therapy has been widely used^1^. Indeed, EGFR controls HNSCC growth, but innate or acquired resistance compromises the efficacy of mono-EGFR-targeted therapy^5^. One of the mechanisms of acquired resistance to EGFR inhibition is activation of alternative pathways such as HER-3^7^. Thus, HER-3-targeted immunotherapy is considered promising for the treatment of HNSCC resistant to EGFR inhibitors.

A multitarget inhibitor of tyrosine kinases may be superior to a mono-specific inhibitor because of the wide coverage of tumorigenic signaling pathways. Dacomitinib is a broad inhibitor of HER-family and downregulates EGFR, HER-2 and HER-4 pathways. However, HER-3, which lacks tyrosine kinase activity, is not affected by this inhibitor^28^. The efficacy of dacomitinib was compared with the EGFR inhibitor erlotinib in a phase III trial, but dacomitinib failed to show selective advantages over erlotinib (unpublished data, clinical trial #NCT01360554). One possible mechanism underlying this failure is the upregulation of HER-3 as we have shown in the present study (Fig. 1B). HER-3 needs to form a heterodimer with either EGFR or HER-2 (both of which are targets of dacomitinib) for activation of downstream signaling^29^,^30^. Recently, c-Met was found to be another partner in HER-3-heterodimerization^8^; therefore, it is possible that HER-3/c-Met signaling promotes resistance to dacomitinib. Thus, targeting of HER-3 makes sense when a mono- or pan inhibitor of HER family is used for HNSCC treatment.

Regulatory T cells (Treg) are a CD4 T cell subset that inhibits antitumor T cells via cytokine secretion and a contact-dependent mechanism^31^,^32^. When evaluating cytokine expression, we found that IL-10 was not produced by HER-3 reactive CD4 T cells, suggesting that Treg cells may not be elicited by HER-3_872−886_ peptide *in vitro*. Because immune suppressive factors (e.g. IL-10, TGF-*b*) in tumor microenvironment would induce Treg, T cell exhaustion or anergy, it is plausible that the combination of HER-3 peptide and Th1-polerized adjuvants such as heat shock protein 70 fragment^33^ are required to completely suppress Treg induction and obtain high-functionalized Th1 *in vivo*. Another reason of the requirement of adjuvants *in vivo* is that IFN-γ is indispensable to induce MHC class II molecules on tumor cells for CD4 T cell recognition^13^. Indeed, we could not get T cell responses against tumor without IFN-γ pretreatment (data not shown). Current standard therapies such as radiation and chemotherapy can be possible cues to induce IFN-γ production followed by antitumor T cell responses^34^.

Recently, precise mechanisms of modulation of immunity by molecularly targeted inhibitors have been discovered. For instance, VEGF increases the number of Treg cells and eliminates CD8 T cells; this finding suggests that a VEGF inhibitor can serve as an activator of antitumor immunity^35^. A BRAF inhibitor downregulates IL-10, which reduces the proliferation of effector T cells and dendritic cells, and increases antigen expression and MHC expression^36^. Similarly, HLA-DR expression was found to be upregulated by the broad inhibitor of the HER family in this study (Fig. 6A). Tumor recognition by HER-3-reactive CD4 T cells is enhanced after the broad inhibition of the HER family, suggesting that such an inhibitor may serve as an immunotherapy adjuvant. According to our previous study, this increased HLA-DR expression may be due to the EGFR inhibition^11^. Further investigations are needed to clarify which downstream pathway of EGFR such as MEK, PI3K or STAT3 is responsible for HLA expression. Although the concentration of pan HER family inhibitor in this study (1 μM) seems to be slightly higher than physiologically relevant concentration in patient serum (0.3 μM)^37^, Engelman *et al*. reported that the effect of this inhibitor was comparable between 0.1 μM and 1 μM^38^ indicating that the physiologically relevant concentration of dacomitinib may result in the same conclusion in the present study.

In general, targeting oncogenic signaling by kinase inhibitors cannot achieve optimal clinical responses because of the primary and acquired resistances such as an activation of alternative pathway^39^. Thus, one would assume that while HER-3 positive tumor is killed by HER-3 reactive T cells, HER-3 negative tumor that activates other signaling pathways such as c-Met, EGFR, or HER-2 can still survive and become a major population. In that case, the epitope peptides that we previously described from these molecules for activating antitumor CD4 T cells should be good targets for immunotherapy^11^,^40^,^41^. Moreover, antigen-targeted immunotherapy has an advantage over molecule inhibitors to induce positive feedback loop for the cancer elimination. Once activated immune cells kill tumor, dead tumor releases a huge amount of tumor antigen in an immune preferential environment followed by polyclonal antitumor CD4 and CD8 T cells activation, so-called antigen spreading^42^. After activation of T cells responding to non-vaccine tumor antigens, HER-3 expression is no more needed to recognize tumor by T cells. Thus, antigen-specific immunotherapy with appropriate adjuvants has a potential to overcome the resistance mechanisms in molecule inhibitors.

In conclusion, our results suggest that HER-3 can induce effective helper CD4 T-cell responses that lead to direct recognition and killing of tumor cells. Furthermore, a broad inhibitor of the HER family may serve as an immunotherapy adjuvant due to the upregulation of HLA-DR on the surface of tumor cells. While *in vivo* studies are required to ascertain the safety and the efficacy of targeting HER-3 as an antigen, the combined strategy involving immunotherapy and a broad inhibitor of the HER family seems to be a promising approach to the treatment of HNSCC.

## Materials and Methods

### Cell lines

Mouse fibroblast cell lines that were transfected with plasmids expressing individual human HLA-DR molecules (L-DR4, −9 or −53) were kindly provided by Dr. Robert W Karr (Karr Pharma, Saint Louis, MO) and by Dr. Takehiko Sasazuki (Kyushu University, Fukuoka, Japan). The cell lines HSC3 (tongue squamous cell carcinoma SCC, DR15/15), HSC4 (tongue SCC, DR1/4, −53), Sa-3 (gingival SCC, DR9/10, −53) and Lu65 (lung large cell carcinoma, DR4/15, −53) were supplied by the RIKEN Bio-Resource Center (Tsukuba, Japan). The HNSCC cell line HPC92Y (hypopharyngeal SCC, DR4/9, −53) was kindly provided by Dr. Syunsuke Yanoma (Yokohama Tsurugamine Hospital, Yokohama, Japan). Tumor cell lines SAS (tongue SCC, DR9/15, −53), Calu-1 (Lung SCC, DR7/14, −53), WiDr (colon adenocarcinoma, DR4/7, −53) and Jurkat (T cell lymphoma, a cell line not expressing HLA-DR) were purchased from the American Type Culture Collection (Manassas, VA, USA). All cell lines were maintained in a tissue culture medium as recommended by the supplier.

### Flowcytometry

Expression of TCR Vβ on CD4 T cell lines was evaluated by flow cytometry using multi-analysis TCR Vβ antibodies (Beckman Coulter) and anti CD4 monoclonal antibody (mAb) conjugated with allophycocyanin (Biolegend, San Diego, CA). Expression of HLA-DR or HER-3 in tumor cells was evaluated by flow cytometry using an anti HLA-DR mAb conjugated with fluorescein isothiocyanate (FITC) (BD Pharmingen, San Diego, CA), an anti-HER-3 Ab conjugated with phycoerythrin (Biolegend), respectively. T-cell viability was assessed using Annexin-V conjugated with FITC (Biolegend) or 7-AAD (Biolegend). Tumor cells were incubated with or without HER-3-reactive CD4 T cells, interferon (IFN)-γ (500 IU/ml, PeproTech, Rocky Hill, NJ), erlotinib (1 μM, Selleck Chemicals, Houston, TX) or dacomitinib (1 μM, Selleck Chemicals, Houston, TX) for 48 h before the flow cytometric analysis. The dead cells and the doublets were eliminated by FSC/SSC gating before the analysis. All data were analyzed on the using Accuri C6 cytometer (BD Biosciences, San Jose, CA).

### *In vitro* induction of HER-3 reactive CD4 T cells

HER-3_872−886_ peptide (KTPIKWMALESIHFG) was used throughout this work as an epitope. This peptide (purity >80%) was purchased from Hokkaido System Science (Sapporo, Japan). The procedure utilized for the generation of peptide-reactive helper T cell lines from Ficoll-Conray-centrifuged peripheral blood mononuclear cells (PBMCs) of healthy humans has been described in detail previously^43^. Briefly, CD14 monocytes were sorted from PBMCs using CD14 MACS microbeads (Miltenyi Biotech, Auburn, CA). CD14 monocytes differentiated into dendritic cells (DCs) after 7 days of culture with GM-CSF (50 ng/ml) and IL-4 (1000 IU/ml) at 37 °C in a humidified incubator with 5% CO_2_. HER-3 peptide-pulsed DCs (3 μg/ml for 3 h at room temperature) were co-cultured with autologous CD4 T cells (purified using CD4 MACS microbeads, Miltenyi Biotech, Auburn, CA) in 96-well flat-bottomed culture plates. Seven days after the peptide stimulation, CD4 T cells were restimulated in individual microcultures with HER-3 peptide-pulsed γ-irradiated autologous PBMCs (3 μg/ml) and 2 days later, recombinant human IL-2 (10 IU/ml) was added. After 2 cycles of restimulation, T cells exhibiting a significant response, by cytokine-release (IFN-γ ELISA kits, BD Pharmingen) to HER-3 peptide-loaded irradiated autologous PBMCs, were cloned by limiting dilution and expanded in 24-well plates by weekly restimulation with peptides and the irradiated autologous PBMCs (3 μg/ml). In short-term culture using PBMCs from HNSCC patients (Stage IV hypopharyngeal, tongue, or oropharyngeal squamous cell carcinoma), PBMCs were stimulated with HER-3 or tetanus toxin_830−843_ peptide (10 μg/ml) for seven days and then restimulated with peptide-pulsed irradiated PBMCs for another seven days. The levels of cytokines in culture supernatant were determined by ELISA kit. The culture medium for these procedures consisted of AIM-V medium (Invitrogen, Carlsbad, CA) supplemented with 3% human male AB serum. All blood samples were obtained after appropriate informed consent (the institutional ethics committee gave approval number #1066).

### Measurement of HER-3-specific responses of CD4 T cells

CD4 T cells (5 × 10^4^/well) were mixed with irradiated antigen-presenting cells (APCs) with or without various concentrations of antigens (peptides or tumor lysates) in 96-well culture plates. APCs consisted of autologous PBMCs (1 × 10^5^/well), L-DR 4, 9 or 53 cells (3 × 10^4^/well), autologous DCs (2 × 10^4^/well) or tumor cell lines (3 × 10^4^/well). Tumor lysates were prepared by five freeze–thaw cycles of 1 × 10^8^ tumor cells, resuspended in the serum-free AIM-V medium, which served as an antigen at 5 × 10^5^ cell equivalents per ml. The tumor cell lines were treated with IFN-γ at 100 IU/ml for 48 h before coculturing to enhance HLA-DR expression. To examine the role of pan HER-family inhibitor in upregulation of the surface HLA-DR molecule, HNSCC cell lines were preincubated with or without dacomitinib (1 μM, Selleck Chemicals, Houston, TX) for 2 h before addition of IFN-γ. Dacomitinib and IFN-γ were removed before the coculture. After 48-h coculture, the culture supernatants were collected to measure antigen-induced IL-4, IL-5, IL-10, IL-17A, and IFN-γ production by CD4 T cells using ELISA kits (IL-4, IL-5, IL-10 and IFN-γ: BD Pharmingen, San Diego, CA, IL-17A: eBioscience, San Diego, CA). The responses of CD4 T cells were blocked by adding an anti-HLA-DR mAb L243 (IgG2a prepared from the supernatant of the hybridoma HB-55 obtained from the ATCC) or an anti-HLA- class I mAb W6/32 (IgG2a, ATCC) at 10 μg/ml throughout the 48 h incubation period to demonstrate HLA-DR restriction.

### Cytotoxicity assays

Cytotoxic activity of CD4 T cells was measured using the colorimetric CytoTox assay (Promega, Madison, WI). This system quantifies the release of lactate dehydrogenase (LDH) from target cells. T cells were mixed with 2 × 10^4^ of target cells at several effector-to-target ratios in 96-well round-bottomed culture plates. After 6 h incubation at 37 °C, the supernatants were collected from each well to measure LDH concentration.

### Immunohistochemistry

Immunohistochemistry staining was performed using the Envision^TM^ HRP System (Dako). Formalin-fixed sections were obtained from colon cancer patients. Samples were boiled in pH6.0 citric acid buffer for 20 min to retrieve antigen and inhibited endogenous peroxidase activity following the manufacturer’s instruction. The sections were then incubated with mouse anti-human HER-3 monoclonal antibody (clone RTJ1/2E11, Merck Millipore) diluted 1:100 for an hour in a room temperature, followed by incubation with an HRP-conjugated secondary antibody (30 min, room temperature) and DAB substrate (5 min, room temperature). The institutional ethics committee approved this study (approval number 977), and the appropriate written informed consents were obtained from all patients providing tissue samples.

### Statistical Analysis.

All data are shown as mean ± standard error of the mean (SEM). Statistical significance of the differences was analyzed by using 2-tailed Student’s t test, and *p < 0.05 was considered statistically significant.

### Ethical considerations

All clinical samples used in this study were handled according to the Ethical Guidelines for Medical and Health Research Involving Human Subjects (Ministry of Health, Labour and Welfare, Japan, 2015). All experimental protocols were approved by the ethics committee of Asahikawa Medical University.

## Additional Information

**How to cite this article**: Kumai, T. *et al*. Targeting HER-3 to elicit antitumor helper T cells against head and neck squamous cell carcinoma. *Sci. Rep*. **5**, 16280; doi: 10.1038/srep16280 (2015).

## Supplementary Material

Supplementary Information

## References

[b1] Bonner J. A. . Radiotherapy plus cetuximab for squamous-cell carcinoma of the head and neck. The New England journal of medicine 354, 567–578, doi: 10.1056/NEJMoa053422 (2006). 16467544

[b2] Vermorken J. B. . Platinum-based chemotherapy plus cetuximab in head and neck cancer. The New England journal of medicine 359, 1116–1127, doi: 10.1056/NEJMoa0802656 (2008). 18784101

[b3] Rosa R. . Sphingosine kinase 1 overexpression contributes to cetuximab resistance in human colorectal cancer models. Clinical cancer research: an official journal of the American Association for Cancer Research 19, 138–147, doi: 10.1158/1078-0432.CCR-12-1050 (2013). 23166225

[b4] Kanda R. . Erlotinib resistance in lung cancer cells mediated by integrin beta1/Src/Akt-driven bypass signaling. Cancer research 73, 6243–6253, doi: 10.1158/0008-5472.CAN-12-4502 (2013). 23872583

[b5] Troiani T. . Increased TGF-alpha as a mechanism of acquired resistance to the anti-EGFR inhibitor cetuximab through EGFR-MET interaction and activation of MET signaling in colon cancer cells. Clinical cancer research: an official journal of the American Association for Cancer Research 19, 6751–6765, doi: 10.1158/1078-0432.CCR-13-0423 (2013). 24122793

[b6] Grovdal L. M. . EGF receptor inhibitors increase ErbB3 mRNA and protein levels in breast cancer cells. Cellular signalling 24, 296–301, doi: 10.1016/j.cellsig.2011.09.012 (2012). 21951604

[b7] Sergina N. V. . Escape from HER-family tyrosine kinase inhibitor therapy by the kinase-inactive HER3. Nature 445, 437–441, doi: 10.1038/nature05474 (2007). 17206155 PMC3025857

[b8] Tanizaki J., Okamoto I., Sakai K. & Nakagawa K. Differential roles of trans-phosphorylated EGFR, HER2, HER3, and RET as heterodimerisation partners of MET in lung cancer with MET amplification. British journal of cancer 105, 807–813, doi: 10.1038/bjc.2011.322 (2011). 21847121 PMC3171021

[b9] Del Sordo R. . HER family receptors expression in squamous cell carcinoma of the tongue: study of the possible prognostic and biological significance. Journal of oral pathology & medicine: official publication of the International Association of Oral Pathologists and the American Academy of Oral Pathology 39, 79–86, doi: 10.1111/j.1600-0714.2009.00815.x (2010). 19691460

[b10] Xia W. . Combination of EGFR, HER-2/neu, and HER-3 is a stronger predictor for the outcome of oral squamous cell carcinoma than any individual family members. Clinical cancer research: an official journal of the American Association for Cancer Research 5, 4164–4174 (1999). 10632356

[b11] Kumai T. . EGFR inhibitors augment antitumour helper T-cell responses of HER family-specific immunotherapy. British journal of cancer 109, 2155–2166, doi: 10.1038/bjc.2013.577 (2013). 24045666 PMC3798972

[b12] Koskinen W. J., Partanen J., Vaheri A. & Aaltonen L. M. HLA-DRB1, -DQB1 alleles in head and neck carcinoma patients. Tissue antigens 67, 237–240, doi: 10.1111/j.1399-0039.2006.00558.x (2006). 16573562

[b13] Watanabe Y. & Jacob C. O. Regulation of MHC class II antigen expression. Opposing effects of tumor necrosis factor-alpha on IFN-gamma-induced HLA-DR and Ia expression depends on the maturation and differentiation stage of the cell. J Immunol 146, 899–905 (1991). 1899098

[b14] Hayashi S. . Six-transmembrane epithelial antigen of the prostate and enhancer of zeste homolog 2 as immunotherapeutic targets for lung cancer. Journal of translational medicine 9, 191, doi: 10.1186/1479-5876-9-191 (2011). 22053850 PMC3219573

[b15] Marshall N. B. & Swain S. L. Cytotoxic CD4 T cells in antiviral immunity. Journal of biomedicine & biotechnology 2011, 954602, doi: 10.1155/2011/954602 (2011). 22174559 PMC3228492

[b16] Hahne M. . Melanoma cell expression of Fas(Apo-1/CD95) ligand: implications for tumor immune escape. Science 274, 1363–1366 (1996). 8910274 10.1126/science.274.5291.1363

[b17] Kurts C. . CD8 T cell ignorance or tolerance to islet antigens depends on antigen dose. Proceedings of the National Academy of Sciences of the United States of America 96, 12703–12707 (1999). 10535986 10.1073/pnas.96.22.12703 PMC23058

[b18] Pollack B. P., Sapkota B. & Cartee T. V. Epidermal growth factor receptor inhibition augments the expression of MHC class I and II genes. Clinical cancer research: an official journal of the American Association for Cancer Research 17, 4400–4413, doi: 10.1158/1078-0432.CCR-10-3283 (2011). 21586626

[b19] O’Sullivan D. . On the interaction of promiscuous antigenic peptides with different DR alleles. Identification of common structural motifs. J Immunol 147, 2663–2669 (1991). 1717570

[b20] Liu X. . A novel kinase inhibitor, INCB28060, blocks c-MET-dependent signaling, neoplastic activities, and cross-talk with EGFR and HER-3. Clinical cancer research: an official journal of the American Association for Cancer Research 17, 7127–7138, doi: 10.1158/1078-0432.CCR-11-1157 (2011). 21918175

[b21] Grivas P. D. . HER-3 in colorectal tumourigenesis: from mRNA levels through protein status to clinicopathologic relationships. Eur J Cancer 43, 2602–2611, doi: 10.1016/j.ejca.2007.08.019 (2007). 17920261

[b22] Clancy-Thompson E. . Melanoma Induces, and Adenosine Suppresses, CXCR3-Cognate Chemokine Production and T-cell Infiltration of Lungs Bearing Metastatic-like Disease. Cancer immunology research 3, 956–967, doi: 10.1158/2326-6066.CIR-15-0015 (2015). 26048575 PMC4527878

[b23] Cerami E. . The cBio cancer genomics portal: an open platform for exploring multidimensional cancer genomics data. Cancer discovery 2, 401–404, doi: 10.1158/2159-8290.CD-12-0095 (2012). 22588877 PMC3956037

[b24] Schuler P. J. . EGFR-specific T cell frequencies correlate with EGFR expression in head and neck squamous cell carcinoma. Journal of translational medicine 9, 168, doi: 10.1186/1479-5876-9-168 (2011). 21970318 PMC3198929

[b25] Datta J. . CD4(+) T-Helper Type 1 Cytokines and Trastuzumab Facilitate CD8(+) T-cell Targeting of HER2/neu-Expressing Cancers. Cancer immunology research 3, 455–463, doi: 10.1158/2326-6066.CIR-14-0208 (2015). 25791067 PMC4556111

[b26] Kayagaki N. . Type I interferons (IFNs) regulate tumor necrosis factor-related apoptosis-inducing ligand (TRAIL) expression on human T cells: A novel mechanism for the antitumor effects of type I IFNs. The Journal of experimental medicine 189, 1451–1460 (1999). 10224285 10.1084/jem.189.9.1451 PMC2193058

[b27] Sawyers C. Targeted cancer therapy. Nature 432, 294–297, doi: 10.1038/nature03095 (2004). 15549090

[b28] Kim H. H., Vijapurkar U., Hellyer N. J., Bravo D. & Koland J. G. Signal transduction by epidermal growth factor and heregulin via the kinase-deficient ErbB3 protein. The Biochemical journal 334 (Pt 1), 189–195 (1998). 9693119 10.1042/bj3340189 PMC1219678

[b29] Tzahar E. . A hierarchical network of interreceptor interactions determines signal transduction by Neu differentiation factor/neuregulin and epidermal growth factor. Molecular and cellular biology 16, 5276–5287 (1996). 8816440 10.1128/mcb.16.10.5276 PMC231527

[b30] Hsieh A. C. & Moasser M. M. Targeting HER proteins in cancer therapy and the role of the non-target HER3. British journal of cancer 97, 453–457, doi: 10.1038/sj.bjc.6603910 (2007). 17667926 PMC2360352

[b31] Montagut C. . Identification of a mutation in the extracellular domain of the Epidermal Growth Factor Receptor conferring cetuximab resistance in colorectal cancer. Nature medicine 18, 221–223, doi: 10.1038/nm.2609 (2012). 22270724

[b32] Chen M. L. . Regulatory T cells suppress tumor-specific CD8 T cell cytotoxicity through TGF-beta signals *in vivo*. Proceedings of the National Academy of Sciences of the United States of America 102, 419–424, doi: 10.1073/pnas.0408197102 (2005). 15623559 PMC544311

[b33] Wang Y. . Stimulation of Th1-polarizing cytokines, C-C chemokines, maturation of dendritic cells, and adjuvant function by the peptide binding fragment of heat shock protein 70. J Immunol 169, 2422–2429 (2002). 12193710 10.4049/jimmunol.169.5.2422

[b34] Lugade A. A. . Radiation-induced IFN-gamma production within the tumor microenvironment influences antitumor immunity. J Immunol 180, 3132–3139 (2008). 18292536 10.4049/jimmunol.180.5.3132

[b35] Terme M. . VEGFA-VEGFR pathway blockade inhibits tumor-induced regulatory T-cell proliferation in colorectal cancer. Cancer research 73, 539–549, doi: 10.1158/0008-5472.CAN-12-2325 (2013). 23108136

[b36] Hu-Lieskovan S., Robert L., Homet Moreno B. & Ribas A. Combining targeted therapy with immunotherapy in BRAF-mutant melanoma: promise and challenges. Journal of clinical oncology: official journal of the American Society of Clinical Oncology 32, 2248–2254, doi: 10.1200/JCO.2013.52.1377 (2014). 24958825 PMC4164812

[b37] Janne P. A. . Phase I dose-escalation study of the pan-HER inhibitor, PF299804, in patients with advanced malignant solid tumors. Clinical cancer research: an official journal of the American Association for Cancer Research 17, 1131–1139, doi: 10.1158/1078-0432.CCR-10-1220 (2011). 21220471 PMC3048920

[b38] Engelman J. A. . PF00299804, an irreversible pan-ERBB inhibitor, is effective in lung cancer models with EGFR and ERBB2 mutations that are resistant to gefitinib. Cancer research 67, 11924–11932, doi: 10.1158/0008-5472.CAN-07-1885 (2007). 18089823

[b39] Song X. . ERBB3-independent activation of the PI3K pathway in EGFR-mutant lung adenocarcinomas. Cancer research 75, 1035–1045, doi: 10.1158/0008-5472.CAN-13-1625 (2015). 25596284 PMC4400867

[b40] Kumai T. . c-Met is a novel tumor associated antigen for T-cell based immunotherapy against NK/T cell lymphoma. Oncoimmunology 4, e976077, doi: 10.4161/2162402X.2014.976077 (2015). 25949874 PMC4404796

[b41] Kobayashi H., Wood M., Song Y., Appella E. & Celis E. Defining promiscuous MHC class II helper T-cell epitopes for the HER2/neu tumor antigen. Cancer research 60, 5228–5236 (2000). 11016652

[b42] Corbiere V. . Antigen spreading contributes to MAGE vaccination-induced regression of melanoma metastases. Cancer research 71, 1253–1262, doi: 10.1158/0008-5472.CAN-10-2693 (2011). 21216894

[b43] Kumai T. . Induction of tumor-reactive T helper responses by a posttranslational modified epitope from tumor protein p53. Cancer immunology, immunotherapy: CII 63, 469–478, doi: 10.1007/s00262-014-1533-z (2014). 24633296 PMC11028558

