# Supplementary material for: Targeting HER-3 to elicit antitumor helper T cells against head and neck squamous cell carcinoma
Source: Sci Rep. 2015 Nov 5;5:16280. doi: 10.1038/srep16280 (PMC4633732; doi:10.1038/srep16280)

Supplementary information (SREP-15-15863A)

Targeting HER-3 to elicit antitumor helper T cells against head and neck squamous cell carcinoma

Takumi Kumai[(1)(2)(3)], Takayuki Ohkuri[(1)], Toshihiro Nagato[(2)], Yoshinari Matsuda[(1)], Kensuke Oikawa[(1)], Naoko Aoki[(1)], Shoji Kimura[(1)], Esteban Celis[(3)], Yasuaki Harabuchi[(2)], and Hiroya Kobayashi[(1)]

(1) Department of Pathology, Asahikawa Medical University, Asahikawa, Japan.

(2) Department of Otolaryngology, Head and Neck Surgery, Asahikawa Medical University, Asahikawa, Japan.

(3) Cancer Immunology, Inflammation and Tolerance Program, Georgia Regents University Cancer Center, Augusta, GA

Takumi Kumai: t-kumai@asahikawa-med.ac.jp

Supplementary Figure 1. Representative TCR Vβ expression on HER-3-reactive CD4 T cell lines.

Representative TCR Vβ expressions on HER-3-reactive CD4 T cell lines are shown. The dead cells and the doublets were eliminated by FSC/SSC gating before the analysis of CD4 and TCR Vβ expression.

Supplementary Figure 2. No effect on T-cell viability in the presence of tumor cells.

HER-3-reactive CD4 T cells (H16 and O3) were cultured with or without tumor cells (Sa-3) for 4h (A) or 16h (B). After culture, CD4 T-cells were harvested and stained with Annexin-V and 7AAD.

Supplementary Figure 3. HER-3 expression on colon cancer and mucosa.

Images of immunohistochemical staining for the expression of HER-3 on the colon cancer and normal colon mucosa (n=5). Colon cancer was in the right side and normal colon mucosa was in the left side of each specimen. Colon cancer highly expressed HER-3 on the cell membrane and cytoplasm except patient 5. HER-3 expression in

normal colon mucosa membrane was weaker than colon cancer.

Supplementary Figure 1

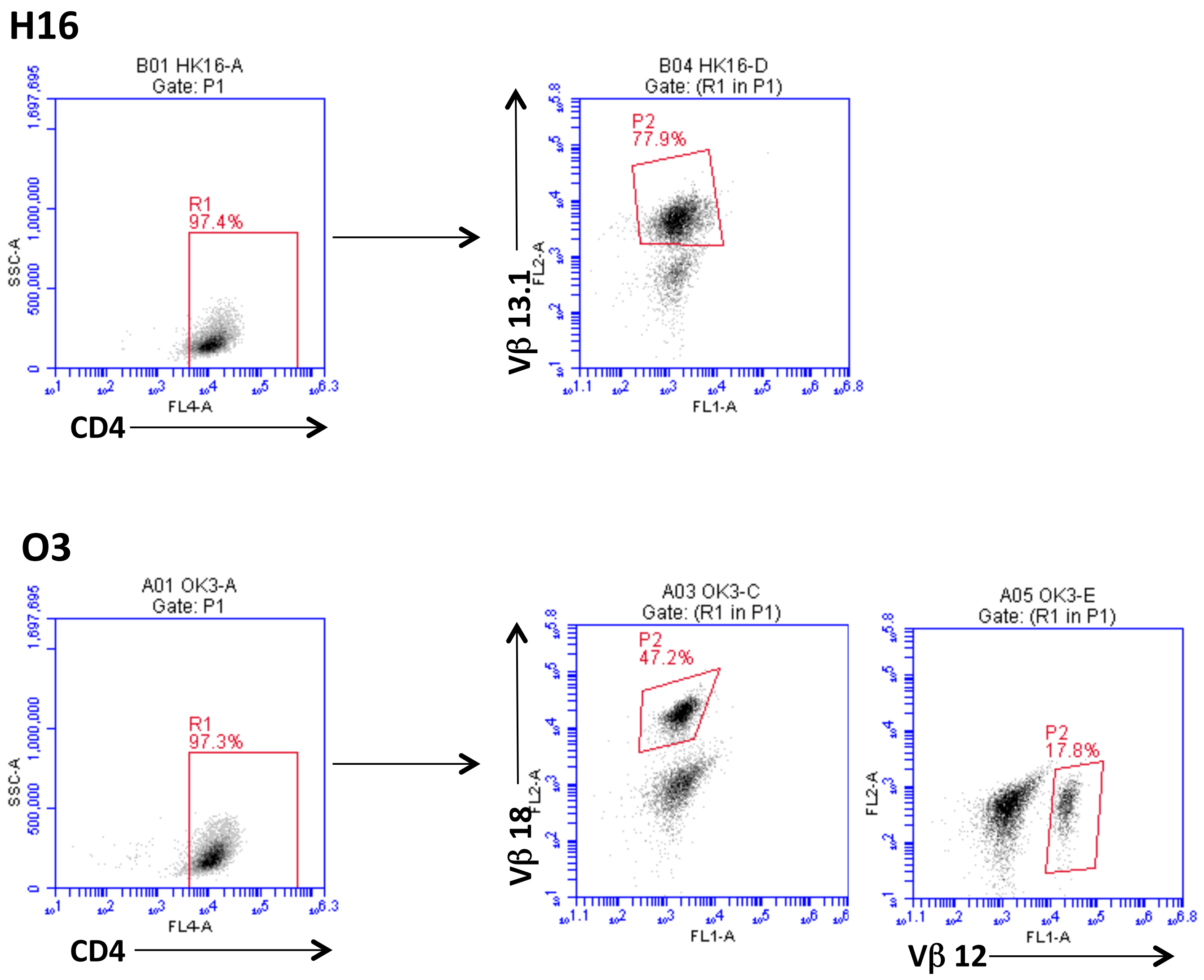

Supplementary Figure 2

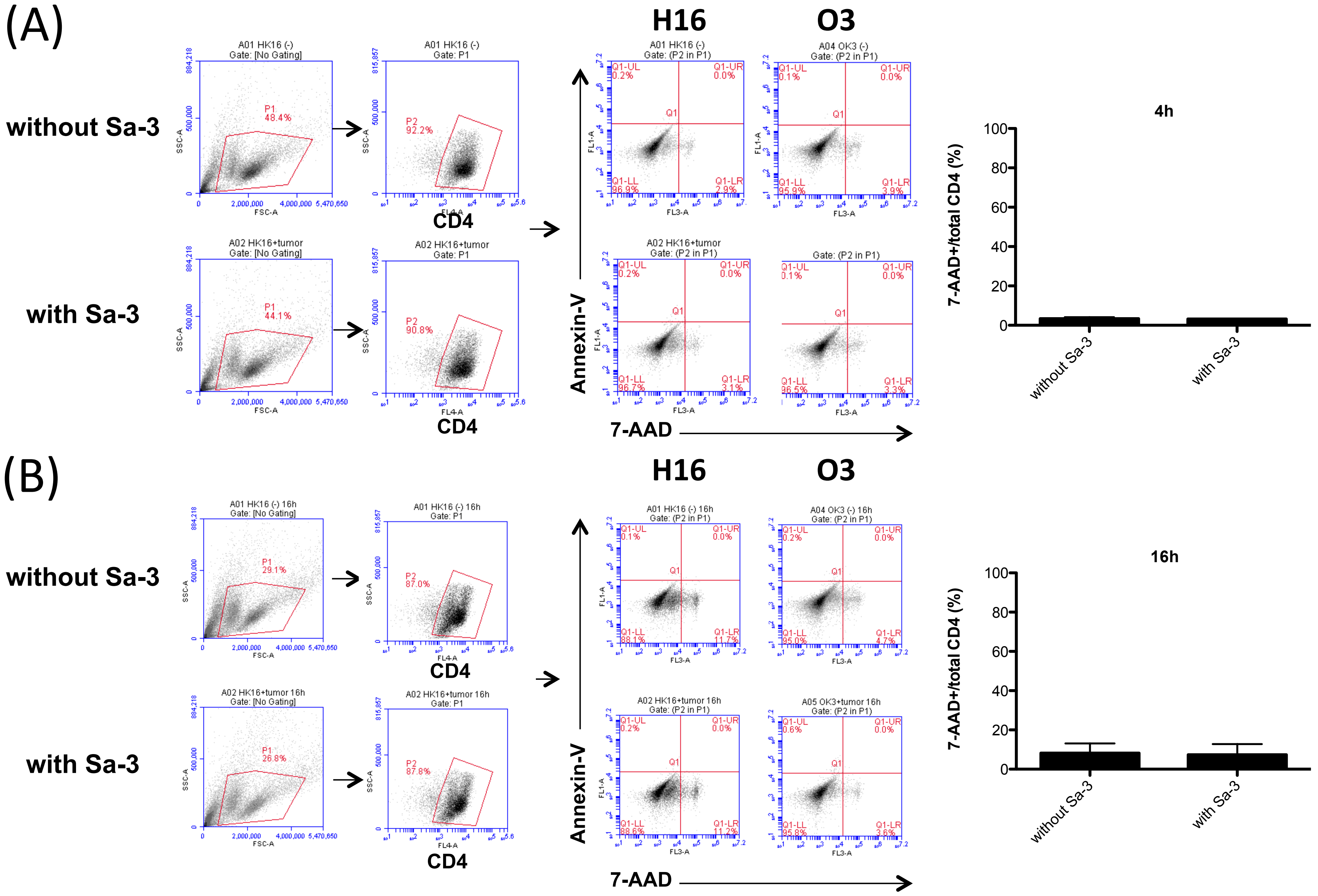

Supplementary Figure 3

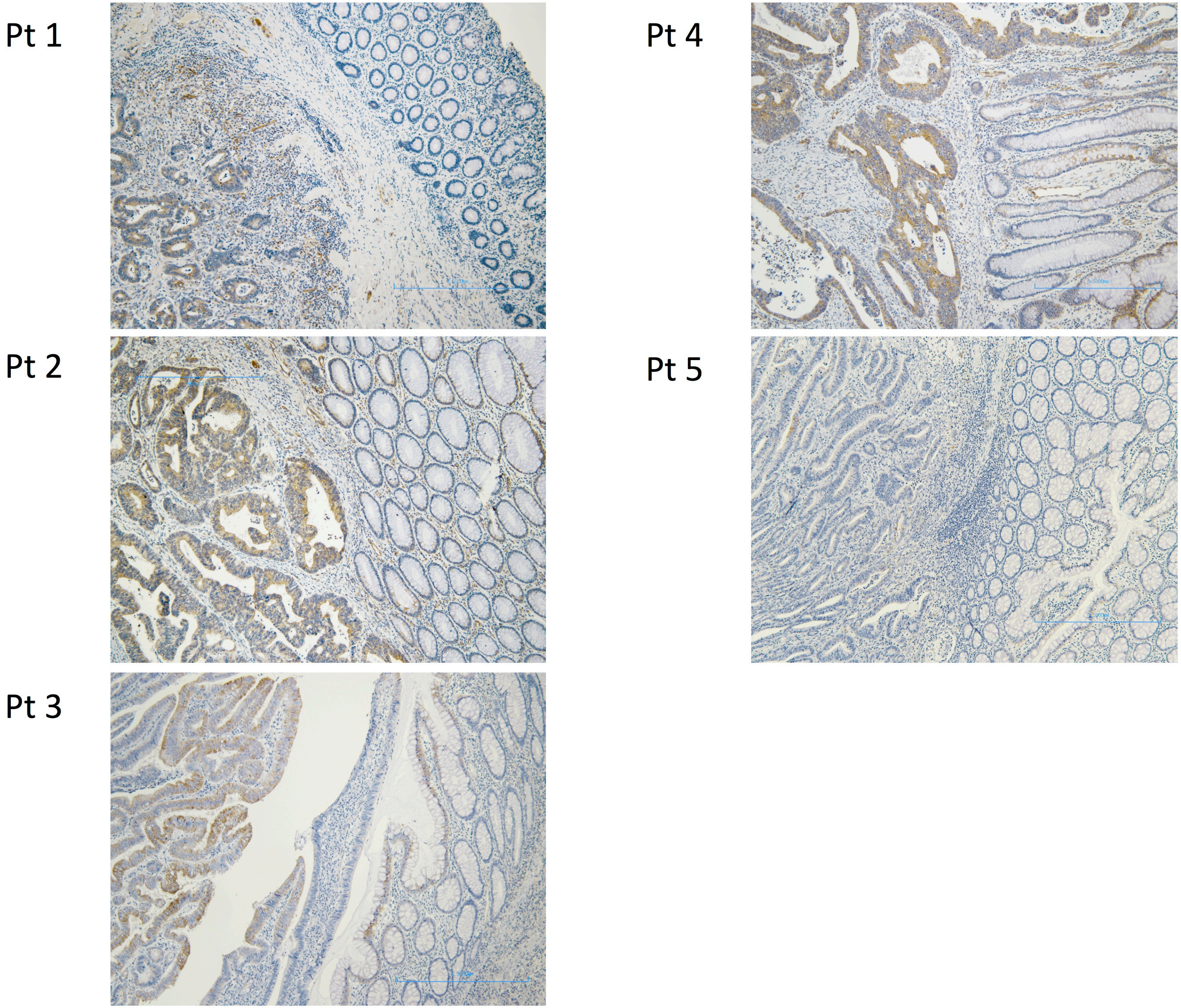

Supplement: Supplementary Information [file srep16280-s1.pdf]
